# Giving Them a Voice: Challenges to Narrative Agency in People with Dementia

**DOI:** 10.3390/geriatrics4010020

**Published:** 2019-02-12

**Authors:** Feliciano Villar, Rodrigo Serrat, Stephany Bravo-Segal

**Affiliations:** Cognition, Development, and Educational Psychology Department, University of Barcelona, 08035 Barcelona, Spain; rserrat@ub.edu (R.S.); sbravo.segal@ub.edu (S.B.-S.)

**Keywords:** person-centered care, dementia, narrative care

## Abstract

In this paper, we argue that the capacity for narrative agency is significantly compromised in individuals with dementia due to at least three factors: (a) Dementia itself, which causes increasing difficulties in constructing and articulating coherent and meaningful stories, and sharing them with others; (b) cultural narratives about dementia, which promote an extremely negative and pessimistic view of those with the disease; and (c) the convergence of these two last factors, which can lead to caregiving interactions that do not support storytelling and can even stop people with dementia from telling stories. We highlight the importance of narrative care, which involves interventions that focus on the person and their unique life narrative. In narrative care, people with dementia are treated not as impaired patients defined by the disease, but as human beings. In doing so, people with dementia can have their own voices back, which is silenced and discredited so many times.

## 1. Introduction

Person-centered care (PCC) has gained increasing relevance in the long-term care of the elderly as an alternative approach to traditional biomedical models that focus on their illnesses and tend to perceive them more as patients than as people [1]. Despite the diversity of conceptual approaches and practices associated with PCC, there is a common core philosophy that considers older adults as unique individuals whose biography and preferences must be always taken into account while delivering quality care [2]. Indeed, one of the main pillars of PCC is to promote a type of care that allows dependent individuals (and particularly those with dementia) to maintain their statuses as people despite the various challenges imposed by the disease.

Therefore, the concept of personhood and its preservation plays a key role in PCC. From a narrative perspective, we perceive human beings as storytellers who build their identities through telling and sharing stories about themselves [3]. Storytelling is not only critical for sustaining personal identity, but also for maintaining autonomy, participating socially, and making their voices and rights as citizens be heard and respected [4,5]. In this paper, we argue that the capacity for narrative agency, defined by Baldwin as “the ability and opportunity to author one’s own narrative” [6], is significantly compromised in individuals with dementia. Difficulties in having a sense of narrative agency can pose challenges in maintaining personal identity and autonomy, producing meaningful social interactions, and even in the exercising of civil rights.

We will discuss at least three factors that contribute to the loss of capacity for narrative agency in people with dementia living in long-term care: (a) Dementia itself, which causes increasing difficulties in constructing and articulating coherent and meaningful stories, and sharing them with others; (b) cultural narratives about dementia, which promote an extremely negative and pessimistic view of those with the disease, the idea of social death [7] being at the very core of the social imaginary; and (c) the convergence of these two last factors, which promotes caregiving interactions that do not support storytelling and can even stop people with dementia from telling stories.

To conceptualize the challenges for narrative agency in people with dementia, an exclusively biomedical-focused model of the disease should be avoided and the role of social and cultural factors in the expression and development of it should be considered [8,9]. In this approach, social interactions with people with dementia are crucial for maintaining their narrative agency. This could not only change our views of the disease, but also the quality and type of relationships we establish with people with dementia. Indeed, what some authors have called narrative care [1,10], defined as “interventions which, in their implementation, focus on the person and his or her unique life narrative” [10], is important for maintaining a sense of narrative agency in those with dementia.

In the following sections, we will first focus on the three main challenges for narrative agency in individuals with dementia and we will conclude by discussing the role that narrative care may have in overcoming these challenges.

## 2. Cognitive Impairments and Their Impact on Narrative Competence

Dementia is a term that describes a set of symptoms associated with an impairment of cognitive functions severe enough to affect the performance of everyday activities. It groups many specific neurodegenerative diseases that develop over time and at a different rates but that, in any case, involve a serious deterioration of functions that are necessary for the elaboration, recuperation, and communication of stories. Thus, dementia profoundly affects individuals’ narrative agency.

Memory problems have traditionally been considered the central and defining element of dementia, especially the most frequent types such as Alzheimer’s disease. Although all mnemonic functions can be affected during the progression of dementia, episodic memory (or the ability to codify, store, and retrieve information) is affected in the early stages of the disease. In particular, memory of recent events is lost earlier than memory of distant, general and autobiographical information [11]. Episodic autobiographical memory of life events, which includes specific temporal and spatial cues, tends to deteriorate earlier than semantic autobiographical memory, which refers to an individual’s general knowledge of certain periods in their life [12]. The impact of this loss is particularly devastating for a person’s ability to tell stories, as autobiographical memories are central for creating and sharing narratives about the self. Indeed, autobiographical memory loss in dementia is one of the key factors contributing to the difficulties in maintaining a sense of personal identity [13]. This is what some authors have called ‘dissolution of the self’, or at least of the part relating to the knowledge of oneself, which occurs at the advanced stages of dementia [14,15].

Deficits associated with dementia, however, are not limited to memory. Linguistic abilities, which are important in creating and sharing narratives, are also seriously affected during the progression of the disease. Semantic-conceptual impairments, affecting both production and comprehension, appear the earliest [16], while syntactic and phonological aspects deteriorate later in dementia [17]. These problems progressively hinder, particularly at the advanced stages of the disease, the individual’s ability to communicate verbally, affecting aspects such as the expression of their needs, their requests for help, or their comprehension of instructions. Narrative agency is therefore highly compromised by the language deficits elicited by dementia. The narratives of people with dementia tend to be repetitive and lack coherence and cohesion. Conversations are affected by sudden changes of subject and sentences may be disconnected from one another, making it difficult for the listener to understand the person with dementia. This is further complicated by the frequent use of generic words, including names (e.g., ‘thing’) and verbs (e.g., ‘to make’), errors in naming, or the use of pronouns with no clear referent [18,19]. This results in a highly repetitive, incoherent, and fragmented narrative containing irrelevant information that is often unintelligible for the listener. Such symptoms vary according to the specific type of dementia, but they tend to be increasingly severe.

However, despite these memory and linguistic impairments, people with dementia may preserve some narrative competence until the advanced stages of the disease [20]. Furthermore, these impairments may result not only from the disease, but also from a social context that does not promote the maintenance of the individual’s narrative agency [21]. We address this important issue in the following section.

## 3. Sociocultural Narratives of Dementia

Although we began the previous section by saying that ‘dementia is a neurodegenerative disease’, it is much more than just that. Dementia is also an object of social knowledge, a set of shared conceptions in narrative form that describes the disease and how it evolves and provides expectations about the behavior of those with the disease [22]. Such shared narratives define our approach to the disease and, consequently, to the people experiencing it. Thus, one key challenge to narrative agency in people with dementia is associated with the place that dementia has in our cultural matrix and the meanings we associate with that condition. Such meanings are reflected, for instance, in the narratives of dementia in social media or in the stories about dementia that we exchange in our everyday conversations, even without direct experience of interacting with people with dementia.

An important part of this knowledge about dementia is built through socialization processes. Conversations, media exposure, or textbooks, among other agents of socialization, not only reflect narratives of dementia, but also help to crystallize them and are the way through which such stories are acquired, internalized, and transformed [23,24]. This knowledge, learned and reproduced in narrative form, serves as a symbolic reference that orients behavior and influences aspects such as the way we express, feel, or act towards certain people or in certain communicative situations [24,25].

Narratives of dementia have been predominantly constructed from the perspectives of the biomedical and scientific communities as well as caregivers. All these perspectives share a negative and pessimistic view of dementia. Biomedical narratives of dementia put narratives of decline at the very core, associating dementia with progressive cognitive impairment, an incapacity for self-care, and loss of autonomy in the advanced stages of the disease [26,27,28]. Caregivers’ narratives about dementia highlight the emotional burden associated with the diagnosis and progression of the disease, as well as the challenges and stress associated with giving care [29,30]. Finally, the narratives of the scientific community emphasize the social, health, and economic burden of dementia. Moreover, they describe scientific attempts to “attack” or “combat” the disease, and use terms such as “epidemic”, “crisis”, or “plague” to describe the prevalence of dementia [11,27].

On top of these narratives of dementia and people with dementia, there are also hegemonic narratives arising from the media that reproduce and legitimize them [30,31]. These media narratives are predominantly partial, negative, and stereotyped, focusing on the most dramatic and extreme aspects of the disease to have a greater impact on the audience [27,32]. Dementia is described in a threatening and apocalyptic way, as a cruel, aggressive, and devastating disease [33,34]. The description of the disease as a “monster” or “invader” that attacks and dominates a person’s mind and body is used often in the media [32,35], reinforcing behavioral patterns that affect the subjective perception of people with dementia, as well as the way in which they are treated and cared for [35,36]. However, in recent decades, there have also been more respectful and heterogeneous narratives of dementia that have raised awareness of the disease and the importance of valuing the needs of those with dementia [22].

From a socio-constructionist perspective, it is argued that societies establish the criteria for identifying and categorizing people, distinguishing between those who are considered normal and those who are not. In this vein, identities are built upon a distinction between ‘us’ and ‘they’, the ‘normal’/’healthy’ and the ‘different’/’sick’. In the case of dementia, the disease is perceived as undesirable, a defect or failure that discredits and reduces people with dementia to a lower social status [22,37]. Central to this narrative is the abovementioned dissolution of the self, which contributes to a process of ‘zombification’ [34] that transforms people with dementia into the living dead, in selfless bodies or in ‘empty shells’ [38,39]. A person with dementia is considered a stranger [38] who has lost their status of ‘being a person’ [30,39] and is experiencing social death [38,39].

These stereotyped narratives promote stigmatization that reinforces the discrimination and exclusion of people with dementia, affecting not only their quality of life, but also those of their relatives and caregivers [34,35].

## 4. Narrative Dispossession in Everyday Interactions with People with Dementia

There is a convergence between the cognitive difficulties in constructing and understanding stories and the presence of cultural narratives emphasizing its exclusively biomedical nature. Both aspects contribute to the perception of dementia as basically a process of dissolution of the person, which has profound implications for the way we establish relationships with people with dementia in everyday life, both in community and institutional settings.

Labeling, which in the case of people with dementia consists of defining the person exclusively on disease terms, is associated with the assumption of a negative narrative of dementia. Just after diagnosis, the person ceases to be a ‘person’ and becomes a ‘patient’, which underlines the presence of dementia as a unifying entity, erasing the biographical and unique qualities that identified the person. The person is not considered an individual anymore and becomes another case of dementia mirroring the nature and evolution of the disease. That is, dementia, as a label, has the power to substitute any previous personal narrative and occupies the core identity of the person.

Once the person has been diagnosed, any behavior (and particularly those that are considered far from normal) is interpreted through the lens of dementia and is vulnerable to being considered a symptom of the disease, something to be controlled or eradicated. Dementia becomes the defining characteristic that gives meaning to their behavior, regardless of their life stories, preferences, needs, expectations, or the actual circumstances that could account for such a behavior from the point of view of the person with dementia [40]. Thus, the person loses their own voice and becomes defined by the disease, or, to be more precise, by the meanings people attach to the disease. Accordingly, the diagnostic label not only overshadows personal circumstance, but can also affect relationships and the way people interact with people with dementia. Frequently, such changes induce a feeling of fear (or even shame) among relatives and friends, who become afraid of the person with dementia behaving aimlessly or unpredictably, or that he or she could violate social norms. Consequently, this contributes to social exclusion and marginalization. People with dementia tend to lose significant social relationships and stop participating actively in the community, which some authors call ‘social death’ [7,41].

The automatic application of such a narrative of dementia is also in line with what some authors, such as Sabat [42,43], have coined as ‘excess disability’, which is defined as the loss of abilities due to factors other than the disease. Thus, the progressive and irreversible deterioration of personal autonomy in dementia promotes a parallel restriction of the opportunities to sustain and reinforce the remaining competences that a person with dementia still has. From this point of view, labeling someone as ‘a person with dementia’ involves losing hope or expectation about the remaining competences of the individual, or about any kind of improvement or learning, quickly leading to a pattern of dependency in which the remaining competences of the person with dementia are ignored. This self-fulfilling prophecy in which a radically negative narrative of dementia leads people to behave in a way that confirms such a narrative is particularly common in institutional settings, where staff interacting with people with dementia work under great pressure to perform certain care assignments [44]. Such dysfunctional interactions affect people with dementia, eliciting disruptive behaviors and expressions of discomfort that are interpreted as a symptom of the disease, thereby reinforcing the initial presumption of a lack of competence [45].

Labeling people with dementia and assuming their disability can lead to poorer communication and a lack of opportunities e.g., [46], particularly for people with dementia living in long-term care institutions. In a study using video recordings of daily life conversations in long-term care institutions for the elderly, Ward et al. [47] estimated that people with dementia spent just 10% of their time interacting with other people, with 75% of this time corresponding to interactions with visitors (mainly close relatives) and the remaining 25% corresponding to interactions with staff or other residents. In a similar study, Doyle and Rubinstein [48] confirmed the ‘communicational neglect’ of people with dementia by staff in care institutions. They observed that most professionals working in long-term care institutions rarely established any communication with people with dementia beyond their assigned tasks, preferring to interact among themselves even when the person with dementia was physically present. According to Doyle and Rubinstein [48], such a situation is possible because the person with dementia is depersonalized and is treated as someone who is non-competent and with less value as a human being.

Depersonalizing people with dementia not only discourages communication and contributes to neglect, but also lowers the quality of the few interactions they have. Thus, caregivers, both in institutional [49] and community settings [50], often address people with dementia in ‘elderspeak’, which consists of speaking more slowly and using inappropriate terms of endearment, exaggerated intonation, simplified syntax, collective pronoun substitution, and questions that indicate a desired response [51,52]. Despite being well-meaning in many cases, the use of elderspeak infantilizes older people by suggesting dependency and a lack of competence, which can contribute to stigmatizing older people and possibly increase disruptive behaviors and resistance to care [53,54].

In institutional settings, contact between residents and staff tend to focus on instrumental care-related tasks, such as activities of daily living, body functions, or health assessments [49]. Thus, in most cases, the interaction consists of getting the resident to cooperate in the task at hand. Consequently, talk is often initiated and directed by the caregiver and is merely instructional, consisting of a set of short standardized imperative sentences and evaluative comments about the task being performed, with few opportunities for the resident to participate actively in the exchange [55]. This way of communicating exemplifies how the professional prioritizes getting through their work over addressing any of the emotional and social needs of the person with dementia that could be met by the interaction. The resident is treated as a frail body and an object rather than a person who is capable of expressing their needs and possessing a unique perspective of the world [56,57].

This type of unbalanced interaction disempowers people with dementia, causing them to lose control over their own lives and become dependent on others who determine their activities, the type and quantity of communication, and the rules governing daily life, particularly (but not exclusively) in institutional settings. In addition, institutional settings generally provide standardized care that prioritizes the efficiency of the staff over the desires and needs of the people with dementia.

Thus, the opportunities for people with dementia to express themselves narratively are dramatically reduced. They have fewer opportunities to get involved in interactions that allow them to construct personal stories, let their voices be heard, and participate in the stories that give meaning to their experiences and link the present situation to their past knowledge and expectations and preferences for the future. Such stories, if they do appear, are controlled by their family or professional caregivers.

Consequently, people with dementia suffer ‘narrative dispossession’ [58], a phenomenon in which they stop elaborating stories or even influence their narrative comprehension of daily experiences. The stories of the people with dementia are hijacked by their caregivers, who assume not just their care, but also the narrative identity of the person with dementia.

## 5. Conclusions and Implications

In this paper, we have reviewed some of the challenges for people with dementia in sustaining their narrative agency, which Baldwin [6] described as the ability to keep on producing stories and the opportunity to express such stories in social contexts. Unfortunately, the ‘ability’ (or disability in this case) aspect has dominated and broadly overshadowed the ‘opportunity’ part of this definition to date. This has happened probably because the disability discourse is consistent with the dominant narrative of dementia that describes it as an unavoidable decline leading to the disappearance of the person and, finally, death. This narrative constructs dementia as an internal process of dissolution, something that happens in the storyteller’s brain that makes them incapable of telling their stories and participating in other people’s stories. Therefore, in accordance with the labeling process described above, any difficulty in narrative production is interpreted as a loss of internal faculties that cannot be ‘fixed’ anymore.

Stressing (dis)ability over opportunity has at least two consequences that further reinforce a total and hopeless decline in narrative agency in people with dementia. Firstly, it contributes to the disregard of the remaining narrative abilities of the people with dementia. These remaining abilities might include non-verbal ways of constructing narratives that can be interpreted by an attentive and proactive audience. In fact, such ‘embodied’ narrative expressions are becoming increasingly important for maintaining narrative agency in the final stages of dementia [59]. Secondly, it releases ‘cognitively intact’ people from the responsibility of sustaining the narrative agency of people with dementia. Storytelling and listening to stories are inherently social acts. People tell stories for and with other people. Thus, the audience has a primary role in stimulating (or hindering) the narrative expression of people with dementia, encouraging them to continue telling stories e.g., [60] and reaffirming their role in the community as active members. Being aware of the narrative challenges that dementia imposes can have important practical implications.

The negative media representation of dementia and of people with dementia has an important effect on the sociocultural narratives of dementia. Although some effort has been made in promoting less hopeless narratives e.g., [61], there is still a long way to go in having a substantially ‘dementia friendly’ (or maybe better, ‘dementia empathetic’) media coverage. Similarly, highlighting the civil rights of people with dementia could also be important, so that they can be thought of and treated as citizens, and not just as diseased individuals or patients. This has been discussed in academic forums in recent years e.g., [62,63,64] and needs to gain momentum in the political arena. To date, associative movement has primarily addressed the many needs of people with dementia and their caregivers. In addition, we should also (a) complement traditional need-based actions with rights-based actions and (b) promote the participation of the people with dementia themselves in such organizations [64]. In this sense, the role of organizations like Dementia Alliance International is remarkable. Such organization is made up by people diagnosed with dementia, and its objective is to provide a unified voice, advocacy and support for rights of people with dementia, honoring its ambitious motto “Nothing about us, without us”.

This paper highlights the importance of narrative care, which involves strategies and interventions focused on recognizing and respecting the unique experiences and stories of every person [65]. At its very core, narrative care aims to treat people with dementia not as impaired patients defined by the disease, but as human beings, both in community and institutional settings. This might appear simple, but, as we have reviewed, it is also sometimes complicated, particularly in care institutions. We should keep talking and share our stories with people with dementia and, at the same time, encourage their narrative agency by supporting and celebrating their stories, whichever way they are expressed.

Caregivers, particularly in residential institutions, should be trained to recognize and facilitate the narrative attempts of people with dementia by using communicative strategies that scaffold and compensate for their increasing difficulties. This would help them create and express meanings regarding their life story and present preferences, enabling them to preserve the self and meet their socioemotional needs. Accordingly, narrative care should be implemented within a more comprehensive cultural change that is in tune with the principles of person-centered care and humanizing care. This might bring benefits to people with dementia and their caregivers, as well as create a more inclusive and decent community by including people with dementia in the narrative fabric of relationships. In doing so, people with dementia can have their voices back, which is silenced and discredited so many times.
